# The prediction of urban growth boundary based on the ANN-CA model: An application to Guangzhou

**DOI:** 10.1016/j.heliyon.2024.e38052

**Published:** 2024-09-19

**Authors:** Lijuan Zhang, Zhenjie Liao

**Affiliations:** a School of Management, Guangzhou Huashang College, Guangzhou, 511300, China; b School of Housing, Building and Planning, Universiti Sains Malaysia, Penang, Malaysia

**Keywords:** Cellular automata, Neural networks, Growth boundary prediction, Guangzhou city

## Abstract

Urban growth boundary (UGB) delineation is critical not only for China's urban planning policies, such as the "three control lines" of the Ministry of Natural Resources, but also for addressing global challenges related to sustainable urban development. This study contributes to the international discourse on urban growth management by developing an innovative artificial neural network-cellular automata (ANN-CA) model, tailored for cities experiencing rapid expansion. Using Guangzhou as a case study, we constructed an impact factor model that incorporates a wide range of factors, including urban spatial terrain, natural environment, current urban land classification, and industrial and economic conditions, along with the layout of modern service networks. The ANN-CA model was then employed to simulate urban spatial expansion and UGB delineation for the year 2030 under various constraints, such as strict protection zones and sustainable development scenarios. Our findings indicate that between 2020 and 2030, Nansha, Panyu, and Zengcheng districts will witness the most significant urban expansion, with respective area increases of 13.81 km^2^, 8.94 km^2^, and 5.8 km^2^, marking them as key growth areas. Furthermore, we propose that future urban expansion in Guangzhou should prioritize the southern and eastern regions, aligning with the city's strategic spatial objectives of "moving east, expanding south, connecting west, and optimizing north." By emphasizing ecological protection and intensive land use, this study provides a robust framework for urban planning in Guangzhou and offers insights applicable to rapidly urbanizing regions worldwide.

## 1 Introduction

As the carrier of urban social and economic activities, the expansion of urban space reflects the phased development and change of cities. Meanwhile, the development process of a city represents also the expansion process of its space. Urban space expansion originates from the endogenous power of urban development. In other words, the determination of urban space growth boundary is an important means of effective governance of urban space expansion. The existing research on urban space expansion is generally based on the changes in remote sensing images of cities over the years. Previous studies used geographic information system (GIS) platforms to build spatial models [1] to analyze and predict the urban space expansion [2,3]. For example, Yin analyzed the urban space expansion of Cairo based on remote sensing images and found that its urban land expansion is based on the sand land in the Nile region [4]. Other scholars studied urban spatial expansion based on urban fractal theory, such as Frankhouser's fractal dimension measurement of urban morphology [5] and White's fractal graphic simulation of urban land use [6].

The urban growth boundary (UGB) is defined as the boundary for urban expansion set at the periphery of a city [7], which requires the city to grow within this boundary [8]. It was first proposed by the "smart growth" movement in the United States. In particular, the delineation of the UGB of Portland, a western city in the United States, has become a classic case [9]. The United States has legalized and taken UGBs as an important means to maintain smart urban growth [10]. Switzerland, India, and other countries have gradually recognized the effectiveness of UGBs in urban planning [11]. Bhatta used GIS and other technologies to simulate the UGB of Calcutta, India [12]. Tayyebi et al. used a neural network and GIS to comprehensively delineate the UGB of Tehran, the capital of Iran [13]. Reasonably delimiting the UGB can improve the utilization rate of urban space and ensure the healthy development of cities by mitigating threats to the ecological environment and providing farmland protection [14,15]. However, the research on UGB in China started late. Zhang Jin first introduced UGB into the Chines context as a tool for urban growth management [16]. Since then, how to scientifically and reasonably delimit UGB has gradually become a research hotspot in China. At present, there are two main methods to delimit the UGB: The first is to conduct land suitability evaluation or ecological security pattern construction through GIS overlay analysis, and then delimit the final UGB by integrating other elements [17,18].The second method is to use an artificial neural network-cellular automata (ANN-CA) model to predict the growth boundary of a city in the future year and then delimit the city growth boundary accordingly [19,20]. While this method considers the internal driving factors of urban expansion, the land suitability assessment method has certain limitations, particularly in addressing the complex and dynamic nature of urban growth. It tends to focus primarily on ecological suitability without fully accounting for the existing population, economic development, and other objective urban conditions. As a result, its application may lead to less accurate predictions and may not fully support sustainable urban development.

In contrast, the ANN-CA method offers a more comprehensive approach by integrating various influencing factors, including both ecological conditions and urban development dynamics. This method helps to balance the protection of the urban ecological environment with the need for expansion, making it particularly suitable for cities with fragile ecological environments and limited construction land, such as river valley cities. Therefore, the use of the ANN-CA model in this study is intended to overcome the limitations of the land suitability assessment method and to provide a more accurate and sustainable delineation of urban growth boundaries (UGBs).

In response to these limitations, recent studies have introduced more advanced models that integrate ecological and landscape considerations. For example, the landscape-driven patch-based cellular automaton (LP-CA) model has been used to simulate urban land-use changes by incorporating landscape patterns into the cellular automaton framework, providing a more nuanced approach to urban growth modeling [21]. Additionally, the Future Land Use Simulation (FLUS) model, which couples the maximum entropy model with cellular automata, has shown promise in predicting future urban land-use changes while also accounting for ecological sensitivity, particularly in scenarios involving urban waterlogging [22]. These models represent significant advancements over traditional ANN-CA models by better addressing the ecological aspects of urban expansion.

In summary, the aforementioned methods in the extant literature have some shortcomings. For example, they only consider the ecological suitability [23] and ignore the existing population, economic development, and other urban objective conditions in the study area [24,25], which is subjective, or they only conduct simulation prediction in terms of quantity and space [26], which has certain limitations. Therefore, following [27], this study calculates the relationship between various influence factors and urban construction land according to the coupling ANN algorithm and uses the CA computational model to simulate the urban land change and growth boundary in the urban area of Guangzhou in 2030.

This study is particularly significant in the context of China's "three lines of control" policy, which includes the ecological protection red line, the permanent basic farmland protection line, and the urban development boundary. These lines are critical for ensuring sustainable urban development, protecting vital ecological spaces, and maintaining food security. By incorporating these control lines into our model, the study provides a more comprehensive and realistic simulation of urban growth, which is essential for informed urban planning and policy-making.

Moreover, the practical significance of this research lies in its potential to promote high-quality urban development and optimize urban spatial layout. By accurately predicting the spatial change and growth boundaries of urban land, the study contributes to the scientific basis needed for urban planners and policymakers to make strategic decisions that align with national and regional development goals. Our results do not only make long-term predictions for the region but also enhance the research on spatial change and strengthen the scientificity and reliability of urban land change simulation.

## 2 Materials and methods

### 2.1 Overview of the study area

Guangzhou is located in the central south of Guangdong Province at 113° 17 east longitude and 23° 8 north latitude, in the south of Chinese Mainland and on the northern edge of the Pearl River Delta. Guangzhou is adjacent to the South China Sea, Hong Kong Special Administrative Region, and Macao Special Administrative Region. Thus, it is China's south gate to the world. Guangzhou is a hilly area. The terrain is high in the northeast and low in the southwest. It has mountains in the north and northeast, hills and platforms in the middle, and alluvial plains in the Pearl River Delta in the south. As the capital and sub-provincial city of Guangdong Province, the core city of the Guangdong-Hong Kong-Macao Greater Bay Area, the economic, cultural, science, and education center of Guangdong Province, and the central city of the 10.13039/100015351Pearl River 10.13039/100002465Delta, the weight of future urban development factors in Guangzhou and the prediction of urban space growth are not only the practical needs of Guangzhou's further development of the “metropolis, big garden, and big channel,” but also will play an important role in supporting the development and planning of the Guangdong-Hong Kong-Macao Greater Bay Area.

### 2.2 Data sources

The data sources of this study are the current land use maps of Guangzhou in 2010, 2015, and 2020, with a spatial resolution of 100 m × 100 m(see Table 1). The land use classification status maps were obtained after spatial registration and reclassification. Land use is divided into five categories: cultivated, forest, water, urban and rural construction, and unused land. In the simulation and prediction of urban expansion of Guangzhou, rural development and impact should be comprehensively considered, as there are few villages with small area, high level of infrastructure and public facilities, small distance from the urban area, and rural land that is easy to convert into urban construction land. Therefore, in the simulation and prediction processes of different scenarios, urban and rural construction land is used as the data source of construction land, In the final delineation of the urban growth boundary, the “erosion” and other methods are used to eliminate the scattered rural construction land that has not been converted into urban construction land. We used the geospatial data cloud to download the topographic and geomorphic data of Guangzhou, the Ecology software to process the data within the urban area of Guangzhou, and the ArcGIS software to extract the elevation data (slope and aspect). The national geographic information resource service was employed to obtain the current urban road data of Guangzhou. In addition, the impact factor data required by the coupling ANN was extracted from Baidu's open source POI big data. Using Python and other software, various impact factor data were effectively obtained and imported into the current land use map in ArcGIS as vector data information. After unifying the coordinate system, the corresponding spatial correction was conducted [28,29].

**Table 1:** Research data sources and resolution.

Name	Data sources	Attribute
Landuse (2000,2015,2020)	Geospatial Data Cloud (GDC)http://www.gscloud.cn/	100x100m
POI data	Baidu's open source POI big data	Feature data
DEM	Geospatial Data Cloud (GDC)http://www.gscloud.cn/	30x30m
Slope	Generated by DEM	30x30m
Aspect	Generated by DEM	30x30m
GDP	Statistical Yearbook	

### 2.3 Identification of impact factors and potential of urban space expansion

**Fig. 1:**
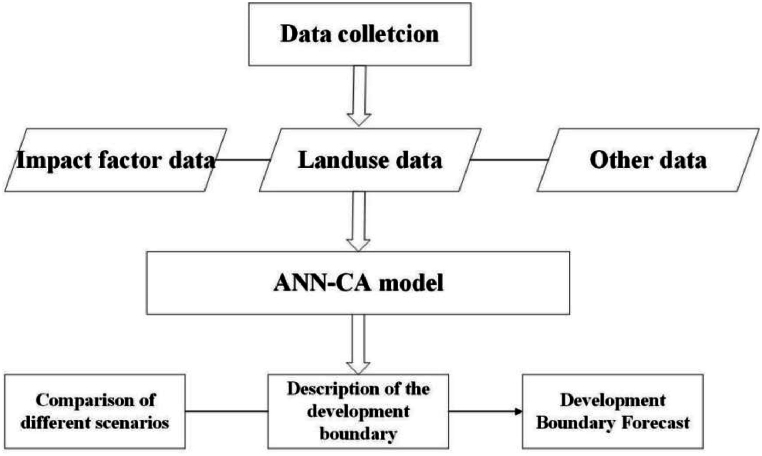
Flowchart of ANN-CA model.

To effectively simulate and predict urban spatial evolution, a comprehensive technical roadmap was developed. This roadmap integrates various data sources, including impact factor data, land use data, and other relevant data, which are then processed through an ANN-CA model. The model allows for a detailed analysis and prediction of urban development boundaries under different scenarios. The following diagram illustrates the technical roadmap employed in this study(As shown in Fig. 1).

#### 2.3.1 ANN-CA simulation

Many spatial impact factors need to be used in the simulation and prediction of urban spatial evolution, thus making it difficult to determine the composition of urban spatial models [30]. Since traditional prediction methods are usually unable to effectively distinguish microscopic spatial variables in the study area [31], it is almost impossible to determine the development direction and evolution characteristics of an urban space system at the microscopic level in the simulation process of urban space. Conversely, the CA model eliminates this disadvantage in the simulation of urban space and can identify the development and development intensity of space unit fields in the evolution system [32].

As a bottom-up simulation process, CA's predictions are based on the interaction between spatial units within the study area. The development possibility and evolution direction of each spatial unit depend on the combination of the development characteristics of surrounding spatial units and their own spatial impact factors. Therefore, the CA model can explain the complexity of urban spatial system development to a certain extent, but it is difficult to obtain logical support in urban spatial evolution under the influence of urban economic and social factors [33].

To address this challenge, it is necessary to introduce real urban spatial constraint factors into the CA model to control the process of urban land change under realistic conditions [34] and increase the reliability of the simulation results [[35], [36], [37]]. Moreover, the extant research shows that combining the ANN algorithm with the dispersion probability of the CA computation model can effectively improve the simulation quality of the CA model in predicting urban spatial expansion and evolution and avoid the negative impacts of traditional prediction methods [38].

In our study, we constructed a detailed flowchart of the ANN-CA model, outlining the structural design, parameter selection, and data processing steps. The urban land use image was used to train the coupled ANN, allowing the ANN-CA model to accurately determine the composition of the urban space model. We also conducted a backtesting analysis using historical data, which demonstrated the model's validity and accuracy. In particular, owing to the comprehensiveness of urban spatial changes, the combination of realistic urban constraint factors and the ANN-CA model can effectively predict the spatial growth and land distribution dynamics of Guangzhou in different realistic situations and provide more scientific and effective theoretical support for the development and planning of Guangzhou's urban boundaries [39].

#### 2.3.2 Establishment of influence factor model based on euclidean distance

**Fig. 2:**
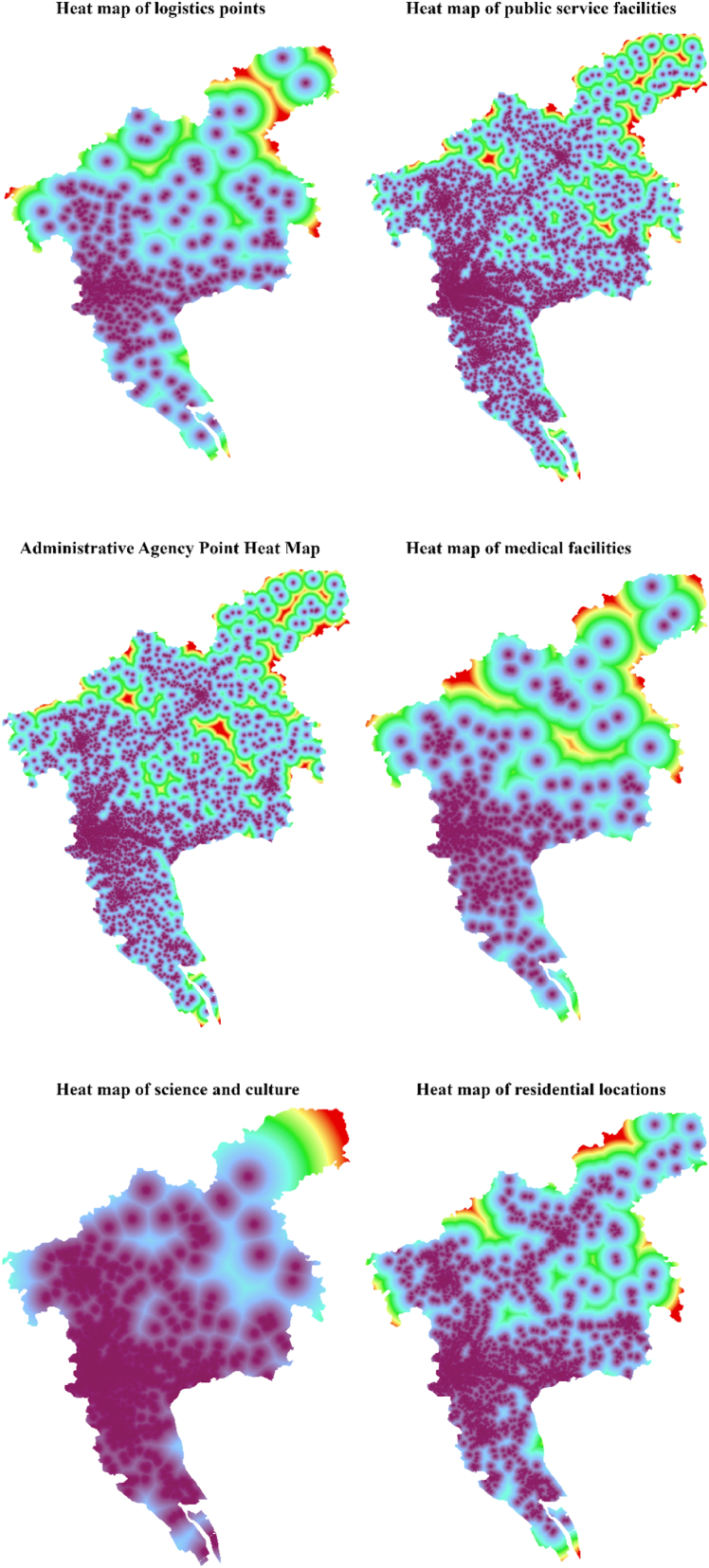

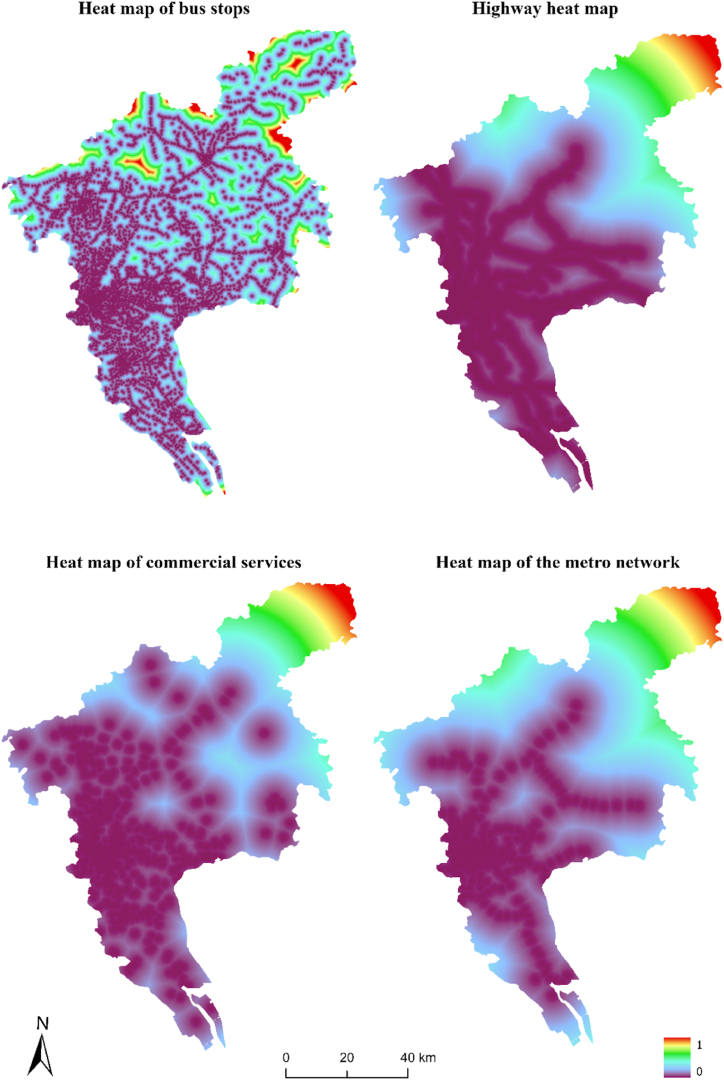
Thermal map of urban influence factors

In addition to traditional urban impact factors, such as urban spatial terrain, natural environment, current urban land classification, and industrial and economic environment factors, we employed big data to extract modern urban impact factors, so that we could more effectively study and evaluate the distribution and characteristics of urban residents' activities and economic and social factors in urban space as well as increase the reliability of urban development potential prediction. The urban impact factors extracted from big data mainly included residential areas in addition to industrial; commercial and public service facilities; science, education, cultural, and entertainment facilities; administrative facilities; tourism and living service facilities; public transport stations; and logistics facilities distribution points. The Euclidean distance was used to process various influencing factors, and their influence heat maps within the urban area of Guangzhou were obtained (Fig. 2).

#### 2.3.3 Identification of urban space development potential based on coupled neural network

The probability of urban spatial growth is based on the development potential of the spatial unit, namely the distribution probability. The ANN can be used to obtain the correlation system of the interactions between urban construction land and various urban impact factors. First, we input the urban impact factors and urban land types into the ANN. Then, we used the ANN to continuously randomly sample data and analyze the interactions between urban impact factors and urban land types, thus generating a correlation system used to explain the relationship between land use types and impact factors. Finally, based on this association system, a classifier was obtained to study and evaluate the contribution weights of different urban spatial development potential levels and urban impact factors to land evolution. The classifier comprises three layers in the following order: input, hidden, and output layer. The last layer is used to calculate the possibility of development. The first step of the classifier is to determine the input of urban land units and impact factors. Each simulation unit contains n variables that will affect land use change and corresponds to n neurons in the input layer. These variables determine the land conversion probability(See Table 2) (see Table 3).

**Table 2:** Contribution weight of influence factors.

Influencing factors	Contribution weight	Influencing factors	Contribution weight
Slope	0.019 582 3	Infrastructure point	0.036 72
GDP	0.078 112 32	Science, education, culture and entertainment	0.033 28
Bus stop	0.132 458	Administrative agency point	0.059 72
National Highway	0.035 448	Settlements	0.022 69
Metro	0.152 448 66	Expressway	0.059 65
Railway	0.075 87	Medical service point	0.035 469 2
Population	0.059 632	Urban arterial road	0.126 73
Logistics point	0.045 83	Tourist spots	0.045 62
Commercial service facilities	0.052 9

**Table 3:** Change of construction land area in each urban area.

	2020(km^2^)	2030(km^2^)
Liwan District	52.26	54.25
Yuexiu District	26.67	27.42
Haizhu District	61.71	64.15
Tianhe District	79.64	80.51
Baiyun District	241.34	246.82
Huangpu District	116.36	119.26
Panyu District	214.22	223.16
Huadu District	194.25	197.87
Nansha District	151.33	165.14
Conghua District	59.61	61.44
Zengcheng District	154.9	160.7

**Fig. 3:**
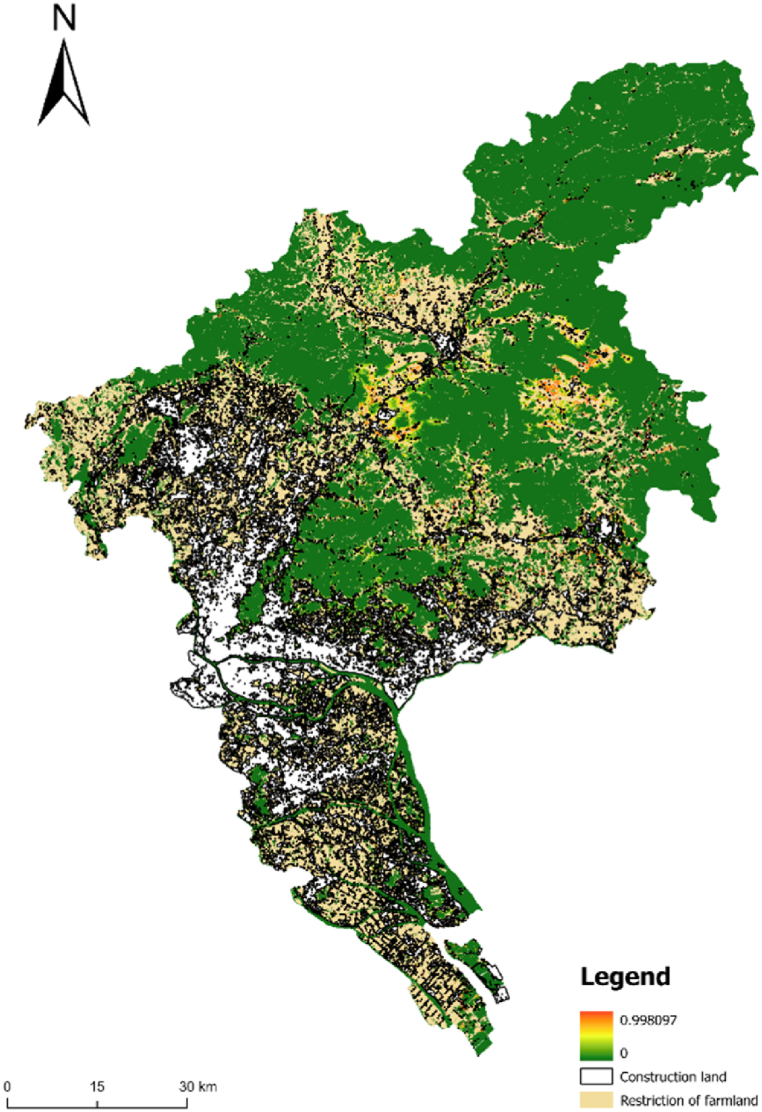
Superposition of basic farmland and urban development probability.

As shown in Table 2, by ranking the importance of each factor from large to small, it can be found that bus stops, urban trunk roads, subways, railways, GDP, and administrative points have a greater impact on construction land. The contribution weights of the top three influencing factors were all greater than 0.12, indicating that the urban trunk roads, bus stops, and subway layout have a high position in the layout of construction land in Guangzhou. The contribution weights of GDP, population, expressways, administrative branches, and commercial service facilities were all greater than 0.050, and the weights were relatively close. Based on the above analysis, traffic impact factors and urban economic development-related factors play an important role in urban development and construction land distribution in Guangzhou. Simultaneously, as an important trade center and tourism city worldwide, the contribution weight of logistics and tourism spots was above 0.045, highlighting that the influencing factors of logistics and tourism resources are critical for the urban development and construction land distribution of Guangzhou. The comprehensive comparative analysis of the regulatory planning map and the urban spatial land conversion probability map shows that the warmer the color is, the greater the possibility that the unit land will be converted into urban construction land, as can be seen in Fig. 3.

## 3 Results and discussion: Guangzhou growth boundary delineation and spatial expansion forecast based on ANN-CA

### 3.1 Land use simulation under different restrictions

In view of the comprehensiveness of urban spatial growth, to effectively predict the spatial growth and land distribution dynamics of Guangzhou in different real-world situations, it is necessary to simulate urban spatial growth by using the combination of real urban constraints and the ANN-CA model. Based on the status quo of land use classification in Guangzhou in 2020, the transformation rule is based on the urban spatial development potential and the contribution weight of urban impact factors to land evolution trained by the classifier. Then, the distribution of urban land use in Guangzhou in 2030 under three different restrictions is simulated. The three limitations of the simulation are as follows.

#### 3.1.1 Strict restrictions

**Fig. 4:**
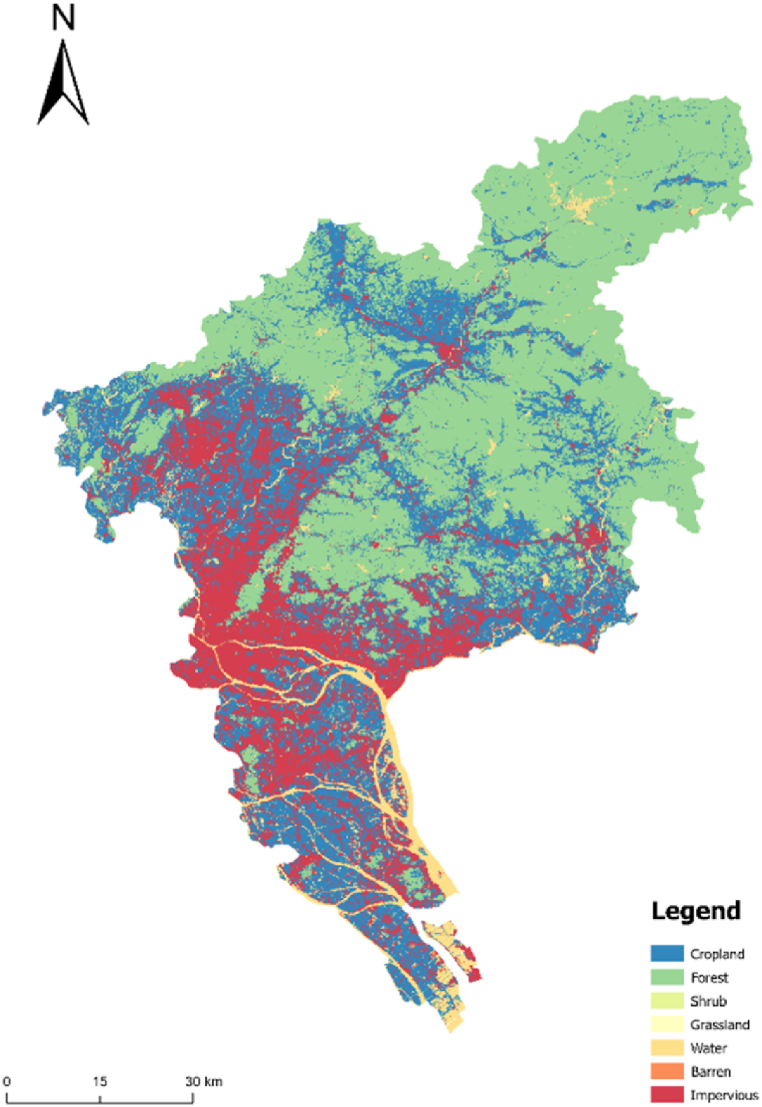
Simulation results under strict constraints.

The land within the primary and secondary control zones cannot be converted into urban construction land. Additionally, the land within the scope of basic farmland protection cannot be converted into urban construction land. Under such conditions and restrictions, the space for urban development is extremely limited. Owing to the limitations of the primary and secondary control zones and the protection scope line of basic farmland, the city can only be limited to the narrow space development between the scope lines; thus, most of the simulated urban land presents a flocculent form (Fig. 4).

#### 3.1.2 Basic limitations

**Fig. 5:**
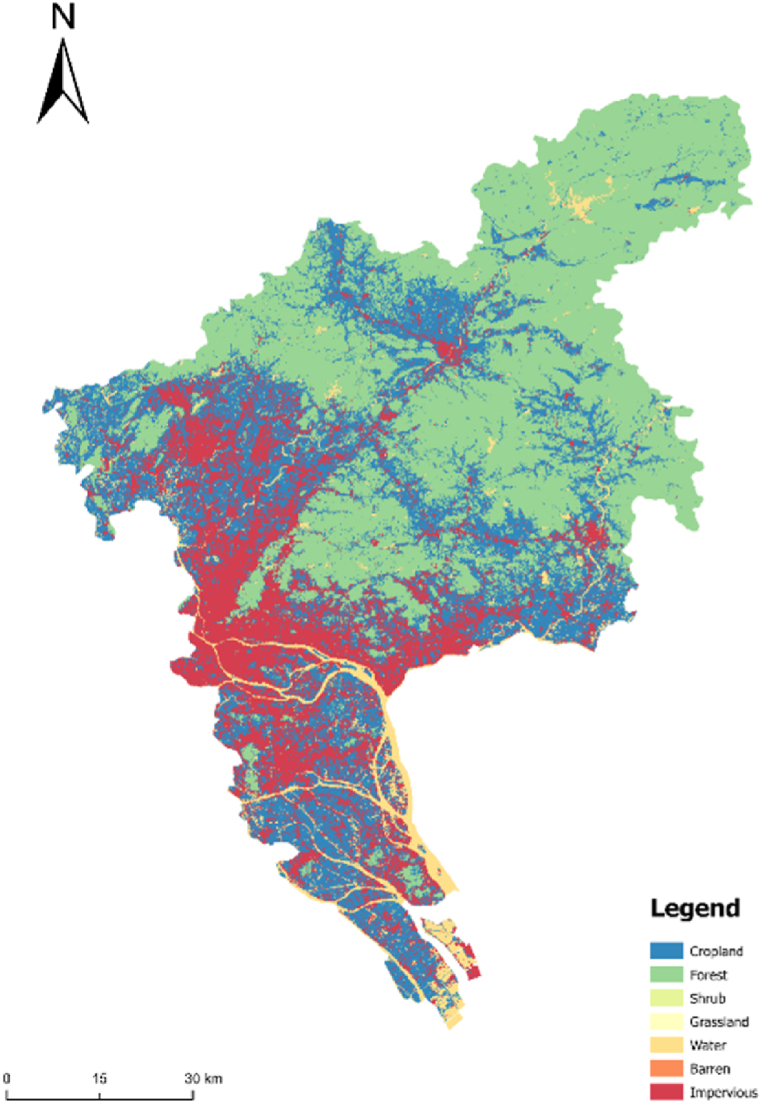
Simulation results of the second-level control zone under unrestricted condition.

The land within the first-level control zone cannot be converted into urban construction land. Additionally, the land within the scope of basic farmland protection cannot be converted into urban construction land. Moreover, the land in the secondary control zone can be converted into urban construction land on the premise of reducing the conversion coefficient. Compared with the urban land simulation under strict restrictions, the urban form under basic restrictions is more complete and regular (Fig. 5).

#### 3.1.3 Control area restrictions

**Fig. 6:**
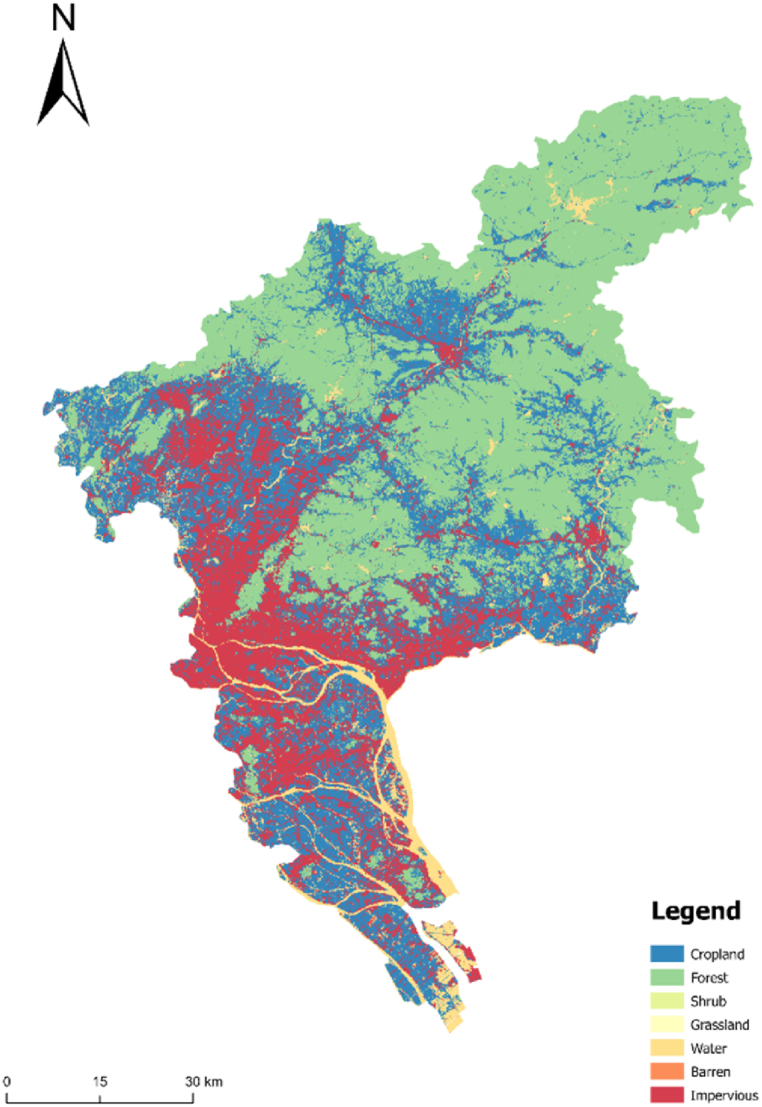
Simulation results of basic farmland under unrestricted conditions.

The land within the primary and secondary control zones cannot be converted into urban construction land. In addition, the land within the scope of basic farmland protection can be converted into urban construction land on the premise of reducing the conversion coefficient. Under such conversion conditions and restrictions, the simulated urban form is complete and smooth, and the urban construction land increases significantly (Fig. 6).

### 3.2 Land use simulation under different realistic scenarios

In reality, the development direction of urban construction will not only be affected by different fixed elements, but also by changes in various national or regional political strategies, urban development planning strategies, and other probability components. Therefore, to a certain extent, the growth scale of a city will not fully comply with the fixed direction of the planning. However, the traditional urban simulation methods cannot eliminate the impacts of these random probability factors on the urban development level. Thus, the CA model can better handle the random effects of these probability factors by virtue of its own randomness. Additionally, the restriction condition is completely composed of rigid land control line and cannot effectively capture the dynamic change of urban land. Thus, it is also necessary to comprehensively consider the flexible restriction factor of urban development in the prediction process. In this study, we simulated the development of land use in Guangzhou under three different realistic scenarios.

#### 3.2.1 UGB under sustainable development scenario

**Fig. 7:**
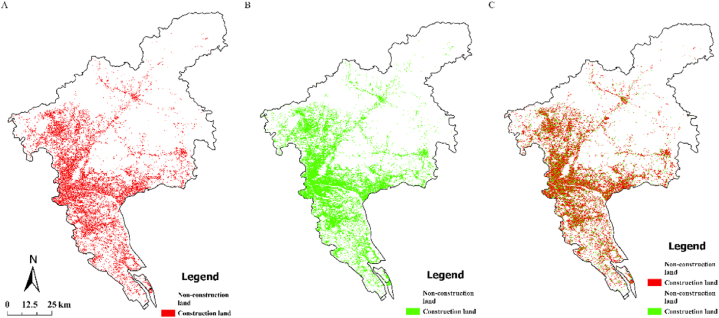
A Distribution of construction land in Guangzhou in 2030 under sustainable development; 7-B Distribution of construction land in Guangzhou in 2030 under scenario of priority development of planning and construction land in core area; Fig. 7-C Overlay of the 2030 construction land distributions under both scenarios, highlighting the differences between the two approaches.

Under the realistic situation of sustainable development and the rigid constraints of basic constraints, the flexible constraints of ecological control and protection are established, and the urban land evolution model that comprehensively considers urban development factors and infrastructure corridors is established. The model comprehensively considers social economy, landform, transportation, and other development factors, which can effectively simulate the dynamic changes in urban space development in the real-world situation of sustainable development and provide a basis for scientific decision-making in urban development. In this model, the land in the first-level control zone cannot be converted into urban construction land. Additionally, the land within the scope of basic farmland protection cannot be converted into urban construction land, and the land in the secondary control zone can be converted into urban construction land on the premise of reducing the conversion coefficient. Moreover, the land in the infrastructure control corridor cannot be converted into urban construction land. Based on the impact on land conversion of relevant constraints extracted from urban development space generated by the above conversion probability, and based on the status quo of land classification in Guangzhou in 2020, an iterative simulation was conducted to draw a simulation map of the distribution of construction land in Guangzhou in 2030 (Fig. 7-A).

Under the control of the realistic scenario based on sustainable development, a small amount of land within the protection scope of the secondary control zone is converted into urban land. Simultaneously, under the comprehensive control and protection of rigid and flexible control conditions, important ecological protection areas, forbidden construction areas, and basic farmland cannot be converted into urban construction land. Under the requirement that the ecological protection land is effectively controlled, this realistic scenario also comprehensively considers the social and economic development. The development of the city is slightly broken in the constrained scope line, but the overall development is good, and the urban form is relatively complete.

#### 3.2.2 UGB under the scenario of priority development of planned construction land in the core area

In this realistic scenario, the future development direction and prospect of Guangzhou are comprehensively considered, and the CA prediction model is improved. That is, it is required to study and evaluate whether the land belongs to the core construction land when identifying the construction land. According to the future development planning of Guangzhou and relevant policies, the land used in the urban construction core area of Guangzhou was extracted and the improved CA model was introduced. If the CA model identified the core construction land under the realistic scenario, we adjusted the corresponding land conversion coefficient to simulate the UGB of Guangzhou under the scenario.

Under this realistic scenario, the land in the secondary control area overlaps with that in the urban core planning, and the land conversion coefficient increases significantly. Compared with the overlapped land, the land conversion coefficient in the secondary control area that does not overlap with the construction land in the core area is significantly reduced. Taking Nansha District as an example, the probability of land conversion to construction land has increased significantly under the realistic situation of core area land.

Moreover, under this realistic scenario, a small part of the land in the secondary control area overlaps with that in urban core planning. At this time, a small part of the land in the secondary control area is converted into urban construction land, the converted construction land is relatively complete and coherent (Fig. 7-B), and the socio-economic development is predicted well. The characteristics of this development are most obvious in the south and east of Guangzhou. Simultaneously, under this realistic scenario, the land within the basic farmland protection, control, and forbidden zones is not converted into urban construction land, and the protection effect is good.

To further highlight the differences between these two scenarios, Fig. 7-C is introduced, which overlays the results of Fig. 7-A and Fig. 7-B. This overlay, represented in Fig. 7-C, visually emphasizes the disparities in construction land distribution between the sustainable development scenario and the core area priority development scenario. By comparing these scenarios, the technical roadmap enables a more nuanced understanding of the impact of different planning strategies on the future urban landscape of Guangzhou.

### 3.3 Simulation integration results and analysis under different scenarios

The simulation of urban land development by the ANN-CA model under diverse realistic scenarios and different restrictions represents the development of future cities under various conditions and theoretical frameworks. These different development scenarios comprehensively consider the complexity and reality of urban space growth, and simulate the integration results and characteristics of Guangzhou's land use under the constraints of sustainable development and core construction land development.

In the scenario based on sustainable development constraints, the spatial distribution and growth of Guangzhou's urban areas are determined with the condition that land within the control lines of basic farmland and primary protected areas is prohibited from being converted into urban construction land. This scenario aims to preserve ecological integrity and ensure long-term sustainability. Conversely, the core area construction scenario presents a more contiguous and developed urban form, with stronger continuity and spatial development. This scenario prioritizes urban expansion in core areas, reflecting a more aggressive approach to urbanization.

**Fig. 8:**
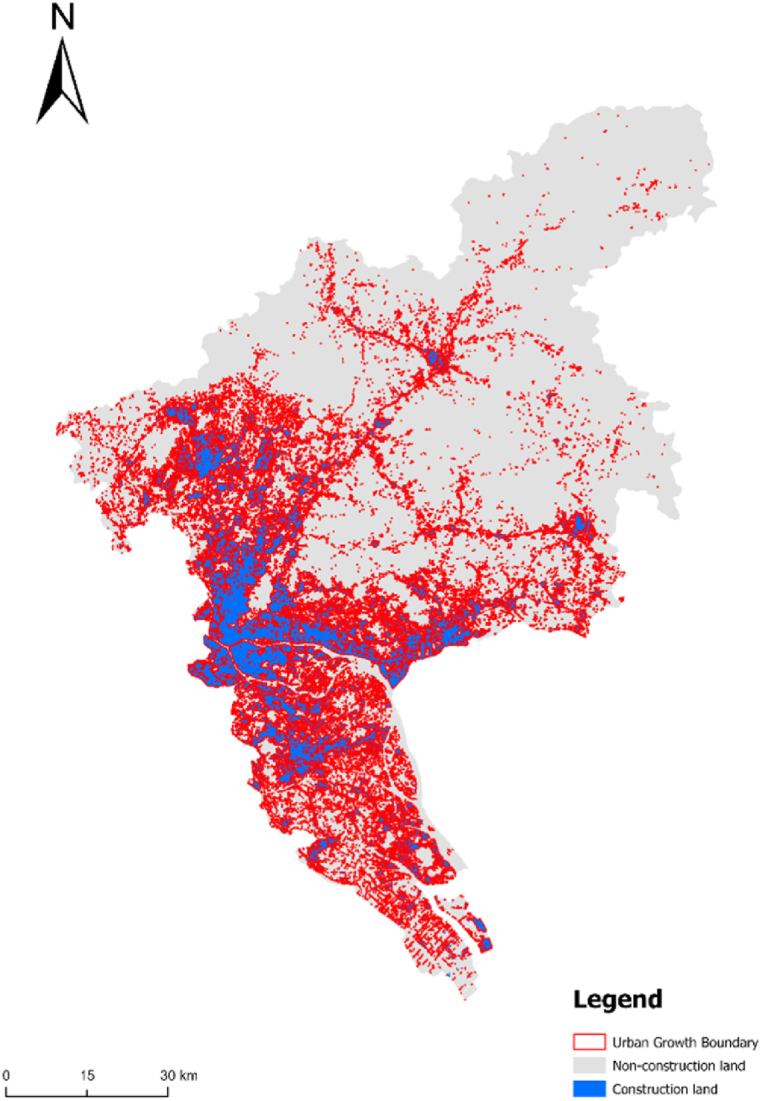
Boundary demarcation and spatial expansion forecast of urban growth in Guangzhou in 2030.

The differences observed in the simulation results under these two scenarios highlight the potential impacts of various urban planning strategies. For instance, in the sustainable development scenario, urban expansion is more constrained, leading to a more fragmented urban growth pattern. In contrast, the core area construction scenario allows for a more cohesive urban expansion, but at the potential cost of ecological sustainability.To refine the simulation results, we used "expansion" and "corrosion" methods to address excessively scattered and fragmented construction land, eliminating rural construction land that had not been converted into urban land. This process resulted in a more practical urban growth boundary (UGB) for Guangzhou with significant reference value (Fig. 8).

According to the integrated area statistics of the simulation boundary under different scenarios (see Table 2, Table 3), from 2020 to 2030, Nansha District, Panyu District, and Zengcheng District will experience the largest urban area expansions, with increases of 13.81, 8.94, and 5.8 km^2^, respectively. These figures highlight the substantial growth potential of these districts, positioning them as key drivers of Guangzhou's urban expansion over the next decade. Overall, Guangzhou shows a strong growth potential, particularly in the southern and eastern regions, reflecting both the strategic developmental focus of the city and the inherent advantages of these areas.

In-depth analysis of these results suggests that the southern direction, particularly Nansha and Panyu Districts, will be key development areas due to several critical factors. Nansha District, for instance, benefits from its favorable terrain and its strategic positioning within the Guangdong-Hong Kong-Macao Greater Bay Area. As a hub for international trade and finance, Nansha's development is not only a local priority but also a key element in the broader regional integration efforts. The district's proximity to major transportation networks, including ports and highways, further enhances its attractiveness for urban expansion and industrial development. Panyu District, on the other hand, is rapidly urbanizing and benefits from its strong connections to Guangzhou's central urban area. The district is evolving into a significant residential and commercial center, capitalizing on its accessibility and the availability of developable land.

The eastern region, especially Zengcheng District, also presents significant expansion potential due to its strategic role as a gateway and a modern industrial cluster area. Zengcheng's development is driven by its designation as a comprehensive gateway in the east of Guangzhou, aiming to integrate urban and rural areas and promote ecological livability. The district's vast areas of undeveloped land provide ample opportunities for large-scale industrial and residential projects, supported by the ongoing improvements in transportation infrastructure, such as the planned Guangzhou Eastern Transportation Hub Center. This hub is expected to enhance the flow of goods and people, further solidifying Zengcheng's role as a key economic and industrial zone in the region.

However, these scenarios come with limitations and uncertainties. The results are based on current policies, land-use plans, and economic trends, which may evolve over time as new policies are implemented, economic conditions change, or unforeseen events occur. For instance, shifts in national or regional development priorities could redirect investment away from these areas, while changes in environmental regulations might impose new constraints on land development. Additionally, technological advancements, such as the rise of smart city technologies or new forms of transportation, could alter the spatial dynamics of urban growth, either accelerating development in certain areas or making others less attractive.

Future research should focus on incorporating more dynamic factors, such as policy shifts, economic fluctuations, population migration patterns, and technological advancements, to further refine the model and improve the accuracy of predictions. This would involve not only updating the model with the latest data but also exploring alternative scenarios that account for potential changes in these factors. By doing so, urban planners and policymakers can be better equipped to anticipate and respond to the challenges and opportunities that lie ahead, ensuring that Guangzhou's urban growth is both sustainable and resilient in the face of an ever-changing landscape.

## 4 Conclusions and policy suggestions

### 4.1 Conclusions

In this study, the ANN-CA model was applied to predict the urban space expansion and evolution of Guangzhou in China. This method was selected because it can effectively identify the micro spatial variables in the study area and determine the development direction and evolution characteristics of urban space systems at the micro level in the simulation process of urban space expansion, avoiding the shortcomings of traditional prediction methods. Using this method, the direction and boundary range of urban space expansion can be determined relatively accurately. Based on the ANN-CA model, this study comprehensively considered the impact factors of urban development and the constraints of rigid control lines under realistic scenarios. Taking 2020 as the base period, we conducted a relatively complete simulation of the urban space expansion and growth boundary of Guangzhou urban area in 2030, and obtained the following conclusions. From 2020 to 2030, Nansha District, Panyu District, and Zengcheng District will have the largest urban area expansion, with the increased areas of 13.81, 8.94, and 5.8 km^2^, respectively, thus demonstrating great development potential in Guangzhou. The main development direction of Guangzhou in the future is in the south and east, which is generally consistent with the spatial strategy of “moving east, expanding south, connecting west and optimizing north” of Guangzhou.

However, it is important to note that urban development is influenced by a multitude of factors, and the expansion direction and boundary of urban space are inherently dynamic. The urban space expansion direction and growth boundary of Guangzhou in 2030, as determined in this study, represent the maximum extent of various types of urban construction land under the simulated scenarios. While these results can guide urban planning and meet the overall development needs of Guangzhou, they must be viewed with caution due to the inherent uncertainties and potential limitations of the ANN-CA model. Factors such as changes in national and regional macro development policies, shifts in the municipal government's priorities, and the evolving needs and opinions of urban residents and stakeholders can all necessitate adjustments to the predicted spatial expansion.

Moreover, the ANN-CA model, while powerful, has its limitations. The model simplifies the complex interactions within urban systems and may not fully capture the impact of unforeseen factors or changes in external conditions. Additionally, uncertainties in the input data, model parameters, and assumptions can lead to potential errors in the simulation results. Therefore, while the model provides valuable insights, it should be complemented with other methods and expert judgment in practical applications. Future research should focus on refining the model to incorporate a broader range of variables and improve its accuracy in predicting urban space expansion under varying conditions.

### 4.2 Policy suggestions

In general, Guangzhou's urban space expansion should adhere to the development model of “moving east, expanding south, connecting west, and optimizing north,” as outlined in the Overall Strategic Concept Plan for Guangzhou's Urban Construction. However, to ensure that these strategies are not only theoretical but also practical and actionable, it is crucial to offer more specific and targeted policy recommendations based on the study's findings and Guangzhou's unique urban dynamics.

Prioritization of Infrastructure Development in Emerging Areas: The study highlights Nansha, Panyu, and Zengcheng districts as areas with significant expansion potential. To support this growth, the municipal government should prioritize the development of critical infrastructure, such as transportation networks, utilities, and public services, in these districts. By improving connectivity and accessibility, these areas can be more effectively integrated into the overall urban framework, facilitating balanced development across the city.

Promotion of Sustainable Land Use Practices: Given the rapid urban expansion projected for 2030, it is essential to implement land use policies that prioritize sustainable development. This includes enforcing stricter regulations on land conversion, protecting green spaces and agricultural land, and encouraging the development of high-density, mixed-use areas to reduce urban sprawl. The promotion of green building standards and the integration of ecological corridors can further enhance the environmental sustainability of urban growth.

Balanced Industrial and Population Distribution: To mitigate the challenges associated with “big city disease,” such as congestion and pollution, Guangzhou should actively pursue policies that decentralize industrial activities and redistribute population growth. This could involve offering incentives for businesses to relocate to emerging districts, as well as developing affordable housing and essential services in these areas to attract residents. The transfer of industries and population should be coordinated with the development of robust public transportation systems to ensure seamless connectivity between new city clusters and the central urban area.

Enhancement of Governance and Public Participation: The success of urban expansion strategies depends on effective governance and the involvement of various stakeholders, including local communities, businesses, and civil society. The city should establish mechanisms for public consultation and feedback, ensuring that urban planning decisions reflect the needs and aspirations of the population. Additionally, fostering partnerships between government agencies, private sector players, and academic institutions can lead to more innovative and context-specific solutions for urban development challenges.

Adaptive Urban Planning Strategies: Recognizing the dynamic nature of urban growth, Guangzhou should adopt a flexible approach to urban planning that allows for adjustments in response to changing circumstances. This could involve regular reviews of the urban expansion model, taking into account new data, emerging trends, and stakeholder input. Adaptive strategies will enable the city to respond proactively to challenges and opportunities, ensuring that urban development remains aligned with long-term goals.

The ultimate objective of these policy recommendations is to facilitate balanced, sustainable, and inclusive urban development in Guangzhou, ensuring that the city's growth not only meets current needs but also anticipates future challenges. By focusing on these targeted actions, Guangzhou can achieve more efficient and effective urban expansion, contributing to the overall well-being of its residents and the sustainable development of the region.

## Data availability

The datasets used and/or analyzed during the current study available from the corresponding author on reasonable request.

## Funding

This study received support from the following sources: a grant from the Guangzhou Huashang College(No.2024HSTS06); a grant from the Guangzhou Huashang College(No.2022HSKT02); a grant from the Guangzhou Huashang College(No.2021HSXK10).

## Ethical approval and consent to participate

The authors declared that they have no known competing financial interests or personal relationships, which seem to affect the work reported in this article. We declare that we have no human participants, human data, or human issues.

## Consent for publication

We do not have any individual person's data in any form.

## CRediT authorship contribution statement

**Lijuan Zhang:** Writing – review & editing, Writing – original draft, Software, Funding acquisition, Data curation. **Zhenjie Liao:** Writing – review & editing, Writing – original draft, Methodology, Formal analysis, Conceptualization.

## Declaration of competing interest

The authors declare that they have no known competing financial interests or personal relationships that could have appeared to influence the work reported in this paper.
